# Review on the impact of heavy metals from industrial wastewater effluent and removal technologies

**DOI:** 10.1016/j.heliyon.2024.e40370

**Published:** 2024-11-15

**Authors:** T.E. Oladimeji, M. Oyedemi, M.E. Emetere, O. Agboola, J.B. Adeoye, O.A. Odunlami

**Affiliations:** a Department of Chemical Engineering, Covenant University, Ota, Ogun state, Nigeria; b Department of Physics, Bowen University, Osun State, Nigeria; c Department of Chemical and Energy Engineering, Curtin University, Malaysia; d Department of Mechanical Engineering Science, University of Johannesburg, South Africa

**Keywords:** Effluents, Heavy metals, Impact, Removal technologies, Wastewater, Water pollution

## Abstract

The incidence of water pollution in developing countries is high due to the lack of regulatory policies and laws that protect water bodies from anthropogenic activities and industrial wastewater. Industrial wastewater contains significant amounts of heavy metals that are detrimental to human health, aquatic organisms, and the ecosystem. The focus of this review was to evaluate the sources and treatment methods of wastewater, with an emphasis on technologies, advantages, disadvantages, and innovation. It was observed that conventional methods of wastewater treatment (such as flotation, coagulation/flocculation, and adsorption) had shown promising results but posed certain limitations, such as the generation of high volumes of sludge, relatively low removal rates, inefficiency in treating low metal concentrations, and sensitivity to varying pH. Recent technologies like nanotechnology, photocatalysis, and electrochemical coagulation have significant advantages over conventional methods for removing heavy metals, including higher removal rates, improved energy efficiency, and greater selectivity for specific contaminants. However, the high costs associated with these advanced methods remain a major drawback. Therefore, we recommend that future developments in wastewater treatment technology focus on reducing both costs and waste generation.

## Graphical abstract

**Figure:**
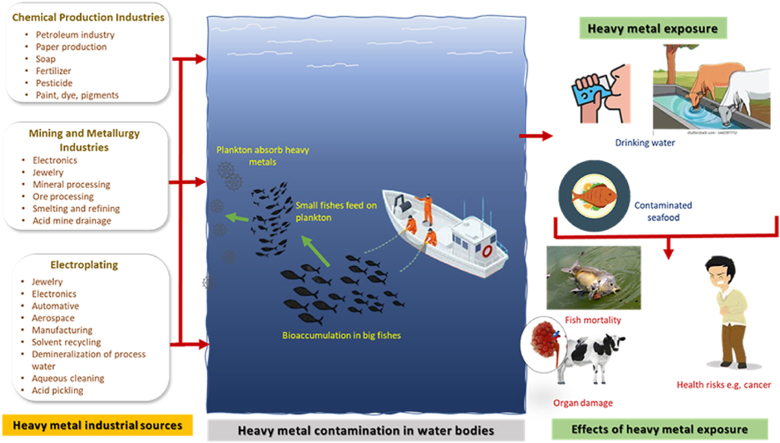

## 1 Introduction

Water, renowned for its universal solvent properties, is extensively utilized across various industrial applications. Nonetheless, the ongoing evolution of human activities, particularly with industrialization, has resulted in the generation of significant volumes of wastewater laden with toxic substances, notably heavy metals. The proliferation of these metals within water ecosystems is ascribed to diverse processes, including industrial activities such as metal plating, electroplating, mining, and battery manufacturing, production of printed circuit boards (PCBs), wood processing, petroleum refining, textile and tannery industry operations [[1], [2], [3], [4], [5], [6], [7]]. Additionally, chemical processes like pipe corrosion and metal equipment deterioration contribute to this phenomenon, alongside direct discharge of heavy metal-containing materials into water bodies and inadvertent spills or leaks.

Heavy metals are persistent non-biodegradable trace elements highly soluble in aquatic environments that tend to accumulate over time. They are inorganic and carcinogenic as they form stable compounds that can migrate throughout the aqueous medium [8,9]. The toxicity of heavy metals can result in inherent health hazards that may give rise to reduced growth and development, cancer, organ damage, nervous system impairment, and, in extreme cases, death when minimal exposure levels are exceeded [10]. Due to the detrimental impact of heavy metals on human health, numerous research studies have emerged recently to address wastewater treatment, including conventional approaches (CA) and advanced technologies (AT) [11]. CA includes adsorption [[12], [13], [14]], chemical precipitation [15,16], flotation [17,18], coagulation/flocculation [19,20], ion exchange [21,22], and electrochemical technologies [[23], [24], [25], [26]]. However, these techniques have demonstrated limitations, including inefficiency in treating wastewater with high acid content, low removal rates, high sludge production needing further treatment, and incurring high disposal costs [27]. While limitations exist with traditional methods, advancements in photocatalysis [28,29], electrodialysis [30,31], genetic engineering [[32], [33], [34]], and microbial fuel cells [[35], [36], [37]] and membrane technologies [38] such as ultrafiltration [[39], [40], [41]], nanofiltration [[42], [43], [44]] and reverse osmosis [45,46] offer promising alternatives. Furthermore, developing new, cost-effective adsorbents with high metal-binding capabilities holds significant potential for improved heavy metal removal [26,47,48]. These modern techniques offer more efficient and economical solutions for heavy metal removal than conventional methods.

Numerous studies have effectively explored the development of effective techniques for removing heavy metals. This review provides an overview of heavy metal contamination in industrial wastewater effluents, focusing on the urgent issue of heavy metal contamination. It sheds light on the different sources of heavy metals within industrial environments. Furthermore, it addresses the hazardous nature of these metals and their adverse impacts on human health and the ecosystem. Techniques like adsorption, flotation, coagulation/flocculation, ion exchange, photocatalysis, electrochemical coagulation, nanotechnology-based filtration, genetic engineering, and microbial fuel cells were explored to identify the most effective methods. By comparing the advantages and limitations of each approach, the review provides valuable insights for future research efforts to develop even more efficient and sustainable strategies for heavy metal removal from wastewater.

## 2 Overview of heavy metal discharge from industrial activities

**Fig. 1:**
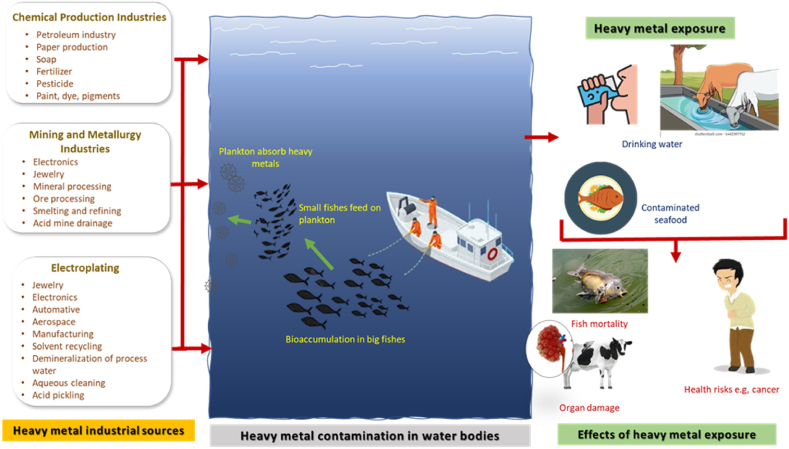
Industrial sources of heavy metals, and the impact of exposure on habitats.

Heavy metals are dense metallic elements with high atomic weights and specific gravities. Heavy metals pose severe threats, causing harm to human health, disturbing aquatic ecosystems, and upsetting the delicate balance of the environment. They are known for their toxicity at certain levels, their long-lasting presence in the environment, and their tendency to accumulate within living organisms. Heavy metals are classified as essential or non-essential depending on their impact in certain concentrations. Essential heavy metals (EHM) are necessary for living organisms in trace amounts, but their excessive presence can result in adverse effects [49], Fig. 1. Fe and Co are components of haemoglobin in the blood. Also, a balanced Zn - Cu ratio can boost immune defense against infection [50]. On the other hand, non-essential heavy metals (NEHM) has no biological functions and are generally considered toxic to living organisms, even at low concentrations [[51], [52], [53]]. Categorizing heavy metals based on their effect helps understand their toxicity and potential impact on human health and the environment. Regardless of this classification, exceeding the permissible levels for any heavy metal can have detrimental consequences.

### 2.1 Specific industries and processes contributing to heavy metal pollution

Heavy metal discharge from industrial activities is a significant source of environmental contamination and poses risks to human health and ecosystems. The research conducted in Refs. [8,54] revealed that industrial activities constitute a major source of heavy metal pollution. Below are critical industries that emit heavy metals.

#### 2.1.1 Chemical production industry (CPI)

The extensive chemical industry encompasses various manufacturing sectors where production relies heavily on chemicals. Examples include the petroleum, fertilizer, pesticide, pulp and paper, paint, dyes and pigment, and soap industries. Heavy metals in the chemical processing industry (CPI) can come from various sources such as raw materials, industrial processes, improper disposal of chemical waste, leaks from underground gasoline pipes, and chemical plant discharges. Major heavy metals associated to these industries include Al, Pb, Hg, Cr, Cu, Fe, Ni, Zn, Hg, Cd, and As [54]. The textile industry often comprises dyes containing heavy metals, aromatic compounds, and non-biodegradable materials [55,56]. Production water from the petroleum industry often contains dissolved heavy metals like cadmium and mercury, posing a significant environmental threat [57]. Processes in paper production such as wood digestion, pulp bleaching, washing, and dewatering activities carried out during processing operations release organic and inorganic substances, tannins, resins, lignin, and other poorly degradable substances such as heavy metals along with their effluents [58].

#### 2.1.2 Mining and metallurgy industry (MOPI)

Mining and ore processing are critical industries that provide the minerals, gemstones, and metals required to manufacture diverse products and materials. Metals are extracted for numerous applications, serving various crucial purposes. For instance, lead is mined for electrical equipment and batteries. Copper is used in construction and electronics, iron forms the basis for automotive and steel components, and Gold and silver in jewelry. These industries involve mineral processing and metallurgy, which are known to produce substantial amounts of heavy metals [59] such as Aluminium (Al), Arsenic (As) lead (Pb), chromium (Cr), copper (Cu), cadmium (Cd), mercury (Hg), and arsenic (As), include lead (Pb), zinc (Zn), Nickel (Ni), and Manganese (Mn). Critical activities in MOPI that lead to heavy metal generation include ore processing (crushing, grinding, and chemical treatment), smelting and refining process, and acid mine drainage [60]. studied the impacts of the steel industry deposit in the urban water system and realized that the city experienced elevated pollution levels from Cd and Zn, mainly due to the significant accumulation of these metals in sediments. Additionally, the steel industry generates significant volumes of wastewater during smelting, Linz-Danowitz, and rolling mill operations. Unfortunately, 90 % of this wastewater is discharged without proper treatment, contaminating water sources [61].

#### 2.1.3 Electroplating industry

Electroplating uses solutions containing metals like chromium, nickel, gold, silver, and zinc to provide a durable and protective finish on various products. This process is used in jewelry, automotive, electronics, aerospace, and manufacturing sectors to improve corrosion resistance, enhance appearance, and ensure electrical conductivity. Wastewater from these operations can contain high levels of toxic heavy metals [[62], [63], [64]]. Processes that result in heavy metal generation include demineralization of process water, solvent recycling, aqueous cleaning, and acid pickling [65].

## 3 Impact of heavy metals in industrial wastewater discharge

Water is used extensively in industrial processes for various functions like cooling, heating, cleaning, dilution, processing, and product transport. However, much of this water is ultimately discharged as wastewater, often containing high levels of heavy metals. Discharging contaminated wastewater directly exposes aquatic life to the dangers of these heavy metals. Bioaccumulating these metals in the food chain can harm humans and animals who consume the water or contaminated fish. Hg, Pb, Cd, As, Cr, Ni, Cu, Zn, Fe, and Mn are some of the major heavy metal pollutants from industrial effluents [8]. Fig. 1 illustrates the industrial sources of heavy metals and how they lead to water contamination, harming habitats.

### 3.1 Arsenic (As)

Arsenic poses a significant environmental risk due to its extreme toxicity, multiple pollution sources, non-biodegradability, and tendency to accumulate in high concentrations [66]. Furthermore, arsenic is expensive to extract and dispose of from the environment, making it a pressing concern for environmental remediation and public health [67]. Arsenic in wastewater primarily occurs in two main inorganic forms: arsenate or Arsenic V (oxidized form of arsenic) and arsenite or Arsenic III (reduced form of arsenic) [68], as seen in equations (1), (2)), respectively. Arsenite is more toxic than arsenate.

(1) $$\text{Oxidation}\;\text{of}\;\text{Arsenite}\;\text{to}\;\text{Arsenate}:As{\left(OH\right)}_{3}+O_{2}\to \;H_{3}AsO_{4}$$

(2) $$\text{Reduction}\;\text{of}\;\text{Arsenate}\;\text{to}\;\text{Arsenite}:H_{3}AsO_{4}+2e^{-}\;\to As{\left(OH\right)}_{3}+{OH}^{-}$$

The redox reactions involve changes in electron count and are influenced by environmental conditions such as oxygen availability and pH.

**Fig. 2:**
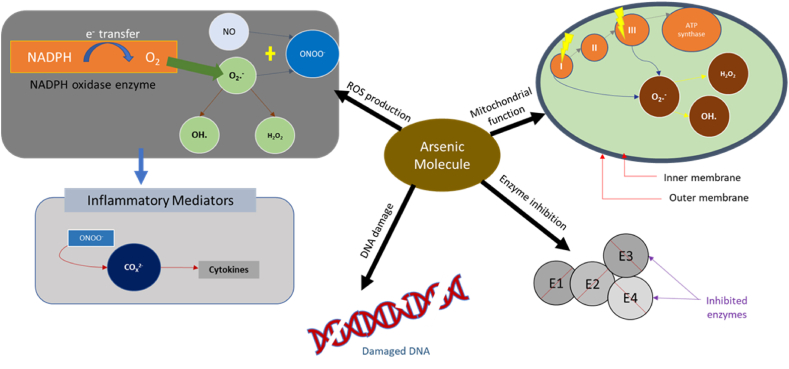
Mechanism of the toxic effects of arsenic exposure.

Arsenic is recognized as a top carcinogen, and the World Health Organization considers arsenic concentrations exceeding 10 parts per billion hazardous [69]. Arsenic's toxic effects on humans are complex and stem from its capacity to disrupt vital biological processes at the molecular level. Arsenic can indirectly produce reactive oxygen species (ROS) through mechanisms like redox cycling and disrupting mitochondrial function. NADPH oxidase facilitates the transfer of electrons to molecular oxygen via the nitric oxide (NOx) catalytic subunit from industrial sources, resulting in the production of reactive oxygen species (ROS) [70]. This generates a potent oxidant called peroxynitrite, which plays a role in upregulating inflammatory mediators, such as cyclooxygenase-2 (COX-2) [71]. It can also inhibit major enzymes in the body, damage DNA, affect the immune system, and result in cardiovascular dysfunction [72]. The illustration in Fig. 2 shows the process of ROS production involving NADPH oxidase, the formation of superoxide anion (O2•-), the conversion to other ROS (H_2_O_2_ and OH•), and the interaction with nitric oxide (NO) leading to the formation of peroxynitrite (ONOO-).

(3) $$\text{NADPH}\;\text{Oxidase}\;\text{Complex}:\text{NADPH}\;\to \;e^{-}\;\to \;O_{2\;}\;\to \;O_{2}^{\cdot -}$$

(4) $$\text{Interaction}:O_{2}^{\cdot -}+NO\;\to \;{ONOO}^{-}$$

Arsenic exposure via contaminated drinking water can have detrimental effects on the renal and reproductive systems. The primary sources of arsenic contamination in water bodies are natural geological activities such as rock weathering and anthropogenic activities such as mining, metal processing, and pharmaceuticals [73]. Several studies have analyzed cases of elevated arsenic concentrations found in rivers, which can be traced back to industrial effluent discharges [[74], [75], [76], [77]]. The consumption of seafood creatures and crops found in areas with high arsenic levels act as pathways for inorganic arsenic to enter the human body. Long-term exposure can lead to acute or chronic poisoning, cardiovascular failure, multiple organ failure, and various cancers [[78], [79], [80]]. While the established Maximum Contaminant Level (MCL) for arsenic in drinking water is 10 μg/L, millions worldwide face exposure exceeding 50 μg/L [81].

### 3.2 Chromium (Cr)

Chromium exists in two major oxidation states, trivalent chromium (Cr (III)) and hexavalent chromium (Cr (VI)), depending on its oxidation state and the surrounding pH of the solution [82,83], as illustrated in equations (5), (6)).

(5) $${Cr}_{2}O_{3}+O_{2}+H_{2}O\;\to \;2H_{2}CrO_{4}$$

(Cr (III)) is oxidized in the presence of oxygen and water to form (Cr (VI)) (chromic acid), which is more dangerous than chromium (III) [6] because it easily finds its way into the body and reducing to Cr (III)). Hence it is referred to as group 1 human carcinogen [84].

(6) $$H_{2}{CrO}_{4}+{3SO}_{2}+{2H}_{2}O\;\to \;{Cr}_{2}O_{3\;}+3H_{2}SO_{4}$$

**Fig. 3:**
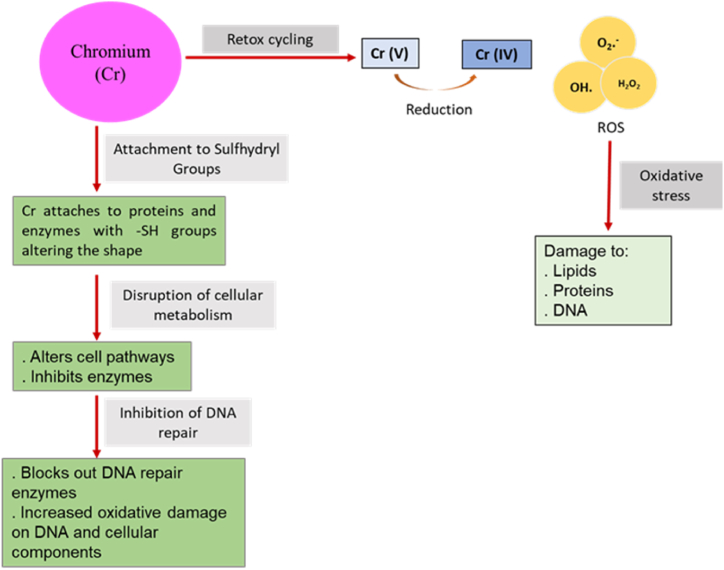
Mechanism of the toxic effects of chromium exposure.

Also, Chromic acid is a strong oxidizing agent which can be reduced to form transient species of tetravalent and pentavalent chromium. This redox cycling produces reactive oxygen species (ROS) such as superoxide anions (O2•−), hydrogen peroxide (H_2_O_2_), and hydroxyl radicals (•OH), leading to oxidative stress that damages lipids, proteins, and DNA [85]. Cr can attach to sulfhydryl (-SH) groups in proteins and enzymes, changing their structure and function. This disrupts cellular metabolism and enzyme activity. For instance, Cr(III) can inhibit enzymes crucial for DNA repair and antioxidant defense, worsening oxidative damage as pictured in Fig. 3.

Depending on the amount absorbed, inorganic Cr(VI) can damage the eyes, red blood cells, skin, respiratory and immune systems [[86], [87], [88], [89], [90]]. Furthermore, chromium exposure can lead to DNA damage and oxidative stress, potentially contributing to the development of cancerous tumors over time [91]. Beyond the severe human health impacts, chromium threatens plant life and soil health [92]. Industrial activities like illegally dumping textile industry effluents, electroplating, dye manufacturing, and tannery wastewater significantly contaminate water bodies [93,94]. Additionally, the combustion of fossil fuels like coal generates ash containing Cr(VI) that can dissolve in rainwater and contaminate waterways. Agricultural practices using certain fertilizers can also spread Cr(VI) in soil and water bodies [95]. Equation (7) shows Cr discharge from Electroplating sources.

(7) $${Cr}_{2}O_{3}+3H_{2}SO_{4}\;\to {Cr}_{2}{\left({SO}_{4}\right)}_{3}+3H_{2}O$$

In this reaction, trivalent chromium oxide is dissolved in sulfuric acid to form chromium sulfate, which can lead to water contamination. The established Maximum Contaminant Level (MCL) for chromium Cr(VI) in drinking water is set at 10 μg/L [96].

### 3.3 Lead (Pb)

Lead (Pb) is a naturally occurring metal with diverse applications. It is a persistent pollutant that remains in the environment for centuries. Due to its ability to form valuable compounds when combined with other elements, acids, or bases, it finds applications in metal processing industries (for corrosion protection), the paint industry, and the battery production industry. Human activities like mining, CPI, and electroplating significantly contribute to lead pollution in the environment [97,98]. Equations (8, (9)) show Pb discharge from Lead-Acid Batteries and painted surfaces, respectively.

(8 $$Pb{SO}_{4}+H_{2}O\;\to \;{Pb}^{2+}+{SO_{4}}^{2-}$$

In this reaction, lead sulfate from battery discharge dissolves, releasing lead ions into wastewater.

(9) $$PbO_{2}+H_{2}O\;\to \;{Pb}^{2+}+O_{2}$$

**Fig. 4:**
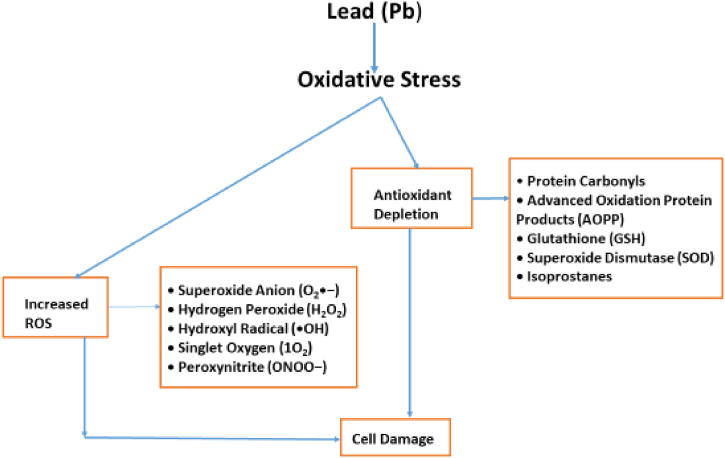
Mechanism of the toxic effect of lead exposure.

Lead induces oxidative stress by producing reactive oxygen species (ROS) and depleting antioxidants like glutathione. This oxidative stress leads to lipid peroxidation, protein oxidation, and DNA damage, resulting in cellular dysfunction and apoptosis as illustrated in Fig. 4. Lead oxide, commonly found in old paints, leaches into water when it comes in contact with rain. The toxic effects of lead in aquatic environments disrupt the central nervous system, membrane structure, and red blood cells of marine life [99]. Lead accumulates in fish gills during respiration in water, posing a threat to human health through fish consumption [100,101]. examined lead pollution in certain rivers and the impact on surrounding communities. While trace amounts of lead exist naturally in the human body (10 μg/dL for adults and 1.4 μg/dL for children), exceeding these levels causes various health problems. In adults, high lead exposure disrupts the reproductive system, causing issues with sperm count and fertility. It also hinders proper fetal growth and development. At severe concentrations, lead poisoning damages the nervous system, kidneys, brain, and bones [97,102]. The recommended Maximum Contaminant Level (MCL) for lead (Pb) in drinking water is a significantly low value of 0.03 μM/L due to its detrimental effects [103].

### 3.4 Mercury (Hg)

Mercury pollution, often associated with gold mining and chemical processes, is a major environmental problem that seriously threatens ecosystems [104]. Equation (10) shows Hg release from chemical processes.

(10) $$Hg{SO}_{4}+H_{2}O\;\to \;{Hg}^{2+}+{SO_{4}}^{2-}$$

**Fig. 5:**
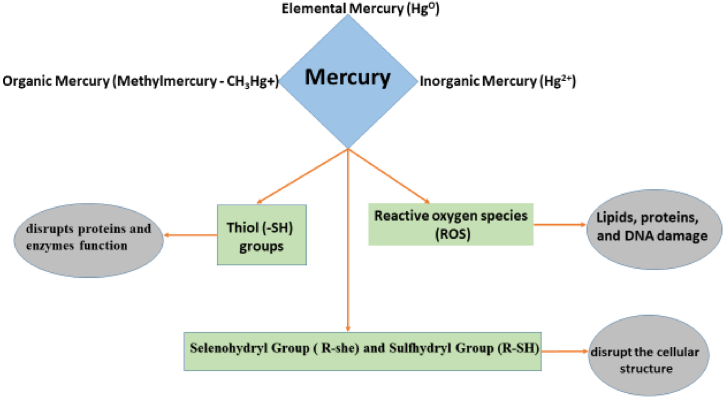
Mechanism of the toxic effect of Mercury exposure.

In this pathway, mercury sulfate, a common byproduct in specific industries, dissolves in water, releasing mercury ions. This toxic metal exists in three oxidation states: Mercury(I), known as mercurous (${Hg}_{2}^{2+})$, Mercury (II) known as mercuric (${Hg}^{2+})$ and elemental Mercury (Hg^0^). Of all forms of mercury, Hg^2+^ is the most prevalent [105]. Fig. 5 shows the mechanism of the toxic effect of mercury exposure

Selenohydryl (R−seH) and sulfhydryl groups (R−SH) are highly reactive with methyl mercury [106] (equations (3.7) and (3.8)) because of the nucleophilic properties of selenium and sulfur atoms.

(11) $$R-seH+{CH}_{3}{Hg}^{+}\to R-Se-Hg-{CH}_{3\;}+H^{+}$$

(12) $$R-SH+{CH}_{3}{Hg}^{+}\to R-S-Hg-{CH}_{3\;}+H^{+}$$

The formation of Selenomethylmercury (R-Se-Hg-CH₃) and Thiomethylmercury (R-S-Hg-CH₃), as seen in equations (11), (12)) can impair the function of selenoenzymes and thiol-containing enzymes, resulting in toxic effects in biological systems.

Mercury poisoning can result in autoimmune diseases, reduced fertility, and neuropsychological effects [[107], [108], [109], [110]]. In the aquatic environment, certain microbial processes convert inorganic mercury into the highly toxic methylmercury (MeHg) bioaccumulates in fish, concentrating further as it travels up the food chain, ultimately reaching humans through fish consumption [[111], [112], [113]]. Studies have documented the link between mercury exposure from fish and neurological effects in children, highlighting the dangers of bioaccumulation [107]. Additionally, research has investigated the detrimental effects of mercury accumulation in the blood [114].

Recognizing these risks, the United States Environmental Protection Agency (EPA) has established a strict Maximum Contaminant Level (MCL) for all forms of mercury in drinking water, set at a very low concentration of 0.002 μg/mL [115].

### 3.5 Copper (Cu)

**Fig. 6:**
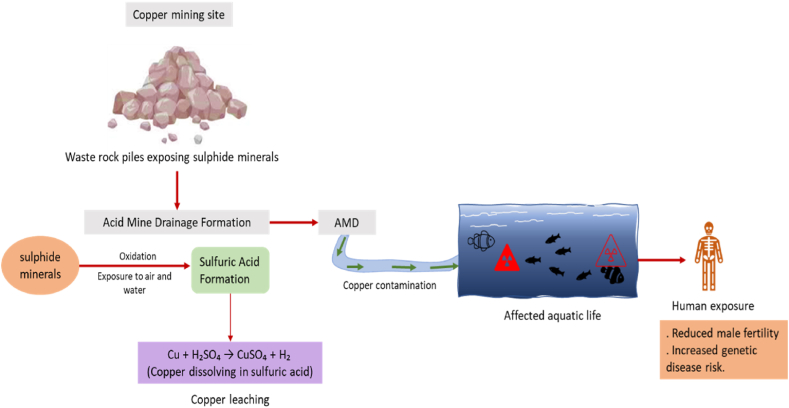
Mechanism of the toxic effect of Copper exposure.

The mining industry is a significant contributor, with acid mine drainage (AMD) releasing copper into waterways [104]. While copper is an essential heavy metal vital for human health, aiding in the growth and regulation of enzymes that assist in catalyzing reactions in metabolic processes, excessive exposure has severe consequences. Studies show that high copper levels can reduce male fertility and increase the risk of genetic diseases [[116], [117], [118]]. Copper finds widespread applications in various industries and agriculture, from alloys and electronics to drug carriers and pesticides, contributing to human and environmental exposure [119,120]. Fig. 6 illustrates the discharge of copper from a mining site, leading to water contamination that adversely affects humans and aquatic life.

(13) $$Cu+H_{2}{SO}_{4}\;\to Cu{SO}_{4}+H_{2}$$

(14) $$Cu\left({OH}_{2}\right)+rain\;water\;\to \;{Cu}^{2+}+{OH}^{-}$$

In eq. (13), copper is dissolved by sulfuric acid, forming copper sulfate, a typical industrial effluent. Also, copper hydroxide, a common pesticide component, dissolves in water, releasing copper ions in eq. (14). The mining industry is a significant contributor, with acid mine drainage (AMD) releasing copper into waterways. Numerous studies have been carried out on the environmental risks associated with copper deposits in rivers and surrounding sediments, highlighting the potential ecological damage caused by the metal [[121], [122], [123], [124]]. The impact of copper smelting on water pollution and the contribution of industrial activities to copper contamination in aquatic environments has also been studied [125,126]. All these studies highlight the detrimental effects of heavy metal pollution from industrial effluents.

### 3.6 Zinc (Zn)

Zinc is essential for humans, plants, and animals. Zinc plays a vital role in numerous biological processes. It contributes to protein folding, DNA synthesis, male fertility, and human growth hormone function. Similarly, plants rely on zinc for proper protein synthesis, chlorophyll production, and overall growth, making it a vital element for a healthy ecosystem [127]. However, It is essential to maintain appropriate intake levels to avoid the detrimental effects of excessive exposure. Excessive zinc in wastewater stems from industrial effluent discharges (Mining, smelting), electroplating, sewage systems, urban runoff, and soil leaching [128]. Equations (15), (16)) show Zn discharge from electroplating and mining processes.

(15) $$Zn+H_{2}{SO}_{4}\;\to Zn{SO}_{4}+H_{2}$$

(16) $$ZnS+O_{2}\;\to ZnO+{SO}_{2}$$

**Fig. 7:**
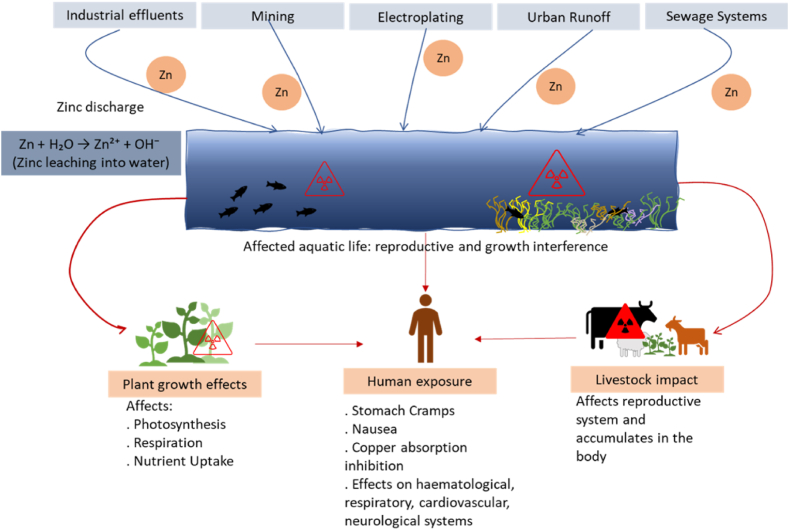
Mechanism of the toxic effect of Copper exposure.

Zinc sulfate and zinc oxide can leach into water, contaminate water, and interfere with the reproduction and growth of certain fish species, threatening the health of aquatic ecosystems. Additionally, it can affect plant growth by disrupting key processes such as photosynthesis, respiration, and nutrient uptake. In humans, elevated zinc levels can hinder copper absorption, which may cause health issues like stomach cramps, nausea, and, in extreme cases, adverse effects on the hematological, respiratory, cardiovascular, and neurological systems [129]. Livestock grazing on zinc-contaminated forage can accumulate high metal levels, exceeding recommended limits and affecting their health [130]. This presents a potential risk to human health if meat products from these contaminated animals enter the food chain. Fig. 7 illustrates zinc discharge from mining and industrial sites, which contaminates water bodies through runoff, affecting plants, humans, and livestock.

### 3.7 Nickel (Ni)

Nickel is a widely distributed transition metal essential nutrient for many microorganisms and plants. However, it can also be a potent toxicant at higher concentrations. Nickel exposure occurs through inhalation, ingestion, or dermal contact. Occupational settings pose a significant risk, particularly in nickel refining, electroplating, mining, and other processes [131]. Equations (17), 18) show Ni discharge from electroplating and mining processes.

(17) $$Ni{SO}_{4}+H_{2}O\;\to \;{Ni}^{2+}+{SO_{4}}^{2-}$$

This reaction represents the dissolution of nickel sulfate, releasing nickel ions into wastewater.

18 $${\left(Ni,Fe\right)}_{9}\;S_{8}+O_{2}+H_{2}O\;\to \;{Ni}^{2+}+{Fe}^{2+}+{SO}_{4}^{2-}$$

Also, Nickel ores, such as pentlandite ${\left(Ni,Fe\right)}_{9}\;S_{8}$, can be oxidized, leading to the release of nickel into water.

The adverse health effects of nickel toxicity range from allergic reactions, such as contact dermatitis, to more severe conditions, which include respiratory issues, cardiovascular and kidney diseases, lung and nasal cancers. Research suggests that oxidative stress, inflammation, and epigenetic changes from nickel exposure can lead to cancer development [131]. Animals exposed to elevated nickel levels experience similar health problems as humans, including respiratory issues, stunted growth rates, reproductive dysfunction, and organ damage [132]. Aquatic organisms are particularly vulnerable due to bioaccumulation and biomagnification within the food web, ultimately affecting the aquatic ecosystem. Nickel enters the environment through natural processes, like weathering and volcanic activity, and human activities, such as industrial emissions, waste disposal, and agricultural practices [132]. Environmental contamination with nickel can occur in soil and water bodies, leading to reduced microbial activity, phytotoxicity (toxicity to plants), and detrimental effects on wildlife populations. pH and temperature further influence nickel's environmental impact and other chemicals' presence [132].

### 3.8 Cadmium (Cd)

Cadmium is a heavy metal that poses severe risks to human health and the environment. Unlike other essential elements such as zinc, cadmium does not provide any known physiological benefit to the human body. Nevertheless, its extensive use in industrial processes has resulted in alarming levels of environmental pollution. Cd contamination in water arises from several sources, primarily related to industrial activities, such as mining, battery manufacturing, and electroplating. Equations 19, 20) show Cd release from electroplating and mining processes [133].

19 $$CdO+H_{2}{SO}_{4}\;\to Cd{SO}_{4}+H_{2}O$$

20 $$CdS+O_{2}\;\to CdO+{SO}_{2}$$

Cadium sulfate $\left(Cd{SO}_{4}\right)$ and Cadium oxide (CdO) are common sources of cadmium in wastewater.

Exposure to cadmium can cause damage to the kidneys and bones. It can also increase the risk of cancer. Exposure to cadmium primarily occurs through contaminated food, water, and air, which accumulates within the body, particularly in the kidneys. This accumulation can trigger a series of harmful health consequences, such as kidney disease skeletal damage due to its interference with bone metabolism, which causes bone demineralization and raises the risk of osteoporosis and, fractures, and cancer. Cadmium has been classified as a Group 1 carcinogen, indicating a well-established connection with the onset of cancers, especially lung and prostate cancer.

Additionally, it can lead to cardiovascular disease and reproductive problems [[134], [135], [136]]. Animals are not immune to the dangers of cadmium. Exposure can lead to organ damage, particularly in the liver and kidneys, immune system suppression, bone lesions and reproductive problems, and bioaccumulation and ecosystem risks [134]. While natural sources like volcanic activity contribute to cadmium pollution, human activities, including mining, metal processing, and phosphate fertilizers, are significant sources. This environmental pollution contaminates soil and water and has negative impacts on plant growth and microbial communities. Reduced plant growth (phytotoxicity) and altered nutrient cycling disrupt ecosystem functions and environmental health [137].

### 3.9 Iron (Fe)

Iron is a heavy metal that is vital to the human body. It is a key component in physiological and biochemical functions, notably oxygen transport and antioxidant defense [102]. However, iron can be detrimental in high doses. Excess iron can contribute to forming free radicals, which are unstable and highly reactive molecules that damage lipids, proteins, and DNA. This free radical generation promotes oxidative stress and increases the risk of carcinogenesis or cancer development [138]. Iron toxicity can result in the formation of hydroxyl radicals, particularly destructive free radicals that can inactivate enzymes crucial for cellular function, initiate a chain reaction of lipid peroxidation damaging cell membranes, depolymerize (break down) essential polysaccharides, and cause DNA strand breaks, ultimately leading to cell death [138]. Fortunately, the toxic effects of iron are generally negligible in low doses. However, when the body lacks sufficient enzymes to neutralize these free radicals, the potential for significant damage increases, especially at high iron intake levels [138]. Iron is widely distributed throughout the environment as it exists in the atmosphere, lithosphere (Earth's solid crust), hydrosphere (water bodies), and biosphere (living organisms and their environment) [102]. Industrial processes like mining, fossil fuel combustion, steel manufacturing, and chemical production can release excess iron into the atmosphere, leading to bioaccumulation (gradual buildup) within living organisms, including humans [102]. Equations 21, 22) show Fe discharge from steel manufacturing and mining processes.

21 $${Fe}_{2}O_{3}+3CO\;\to 2Fe+3{CO}_{2}$$

22 $$FeS_{2}+O_{2}+H_{2}O\;\to \;{Fe}^{3+}+{SO}_{4}^{2-}$$

Equation (21) illustrates the reduction of iron oxide with carbon monoxide in steel manufacturing, leading to potential iron contamination in wastewater, while equation (22) shows the oxidation of iron pyrite, resulting in iron ions and sulfate, which can leach into water.

Exposure to iron can cause damage to the central nervous system, blood composition, lungs, liver, kidneys, and other vital organs. This damage can manifest as physical, muscular, and neurological degenerative processes that mimic certain diseases like Parkinson's and Alzheimer's [102]. High iron concentrations in water bodies can lead to eutrophication, affecting aquatic life and water quality [139].

## 4 Wastewater treatment technologies

The effectiveness of heavy metal remediation technologies depends on several factors, including temperature, pH levels, contact time, amount of adsorbent used, and the initial concentration of metal ions in the wastewater [9,140]. These contaminants pose a significant threat, necessitating treatment to comply with the established Maximum Contaminant Level (MCL) standards that regulate the toxicity of industrial effluents, as outlined in Table 1. Ideally, the treatment methods chosen should be well-suited, appropriate, and adaptable to local conditions while ensuring compliance with MCL standards [141].

**Table 1:** Effects of heavy metals and MCL-acceptable discharge levels.

Heavy metal	Effects on humans	Effects on aquatic life	Effects on the environment	Industrial effluent source	MCL (mg/L)	Ref
Arsenic	Skin lesions, various cancers (lung, bladder, skin), cardiovascular issues, diabetes, developmental problems in children	Bioaccumulation through the food chain, reduced growth and reproduction, organ damage, death	Contamination of soil and water, disruption of microbial activity, reduced plant growth	Wood processing industry Mining	0.050	[73]
Cadmium	Kidney damage, bone demineralization (leading to osteoporosis), increased risk of lung and prostate cancer, potential links to cardiovascular disease and reproductive problems	Organ damage (liver, kidneys), immune system suppression, bone lesions, reproductive issues, bioaccumulation	Pollution, contamination of soil and water, reduced plant growth, and altered nutrient cycling	Paint industry Electroplating	0.01	[8,141]
Chromium	Respiratory problems (irritation, ulcers), gastrointestinal issues (diarrhea, nausea, vomiting), dermatitis, various cancers	Reduced growth and reproduction, behavioral changes, death at high concentrations	Soil and water pollution	Petroleum refining Textile industry Tannery industry	0.05	[8,89,93]
Copper	Liver damage (cirrhosis), stomach ulcers, intestinal bleeding, neurological problems (in extreme cases), genetic disorders (Wilson's disease)	Disrupts vital physiological processes can be lethal at high concentrations	Water contamination depends on water chemistry	Electroplating Mining Agriculture	0.25	[104,116,117]
Mercury	Neurological damage (impaired memory, tremors, coordination problems), developmental issues in children, increased risk of heart disease	Bioaccumulation through the food chain, reduced growth and reproduction, nervous system damage, death	Contamination of air, water, and soil	Mining	0.00002	[107,108,110]
Nickel	Allergic reactions (dermatitis), respiratory problems (irritation, fibrosis), increased risk of lung and nasal cancers	Bioaccumulation, reduced growth, and reproduction can be lethal at high concentrations	Contamination of air, water, and soil	Petroleum refining PCB manufacturing	0.20	[8,141]
Zinc	While essential in low doses, high intake can lead to nausea, vomiting, stomach cramps, headaches, and fever	High concenrations disrupt enzyme function and reproduction	Environmental zinc pollution	Electroplating	0.80	[8,141]
Lead	Kidney damage, nervous system damage (impaired learning, behavior problems), anemia, high blood pressure, increased risk of cancer	Disrupts growth and development, reduces enzyme activity, and can be lethal at high concentrations	Persists for a long time in the soil and sediments	PCB manufacturing Electroplating Mining		[97]

### 4.1 Common treatment methods for removing heavy metals from industrial wastewater

#### 4.1.1 Flotation

One method for removing heavy metals from wastewater is flotation. It relies on gravity and the interaction between the metal ions and special molecules called surfactants. These surfactants attract the metal ions and adhere to the surface of rising air bubbles. The bubbles then form a froth on the water's surface, which can be skimmed off, thereby removing the metals from the solution [142]. This flotation technique was initially used in mining to recover valuable elements, but it can be adapted for wastewater treatment [143]. Optimizing flotation performance hinges on several key parameters which include: using smaller bubbles to increase the surface area for metal attachment, employing collectors to enhance metal particle hydrophobicity, ensuring proper mixing for uniform reagent distribution and effective bubble-particle contact, and selecting the appropriate method for efficient metal removal [144,145]. Different flotation methods are used for heavy metal removal, including ion flotation, sorptive flotation, and hybrid flotation [146].

i. Ion flotation

**Fig. 8:**
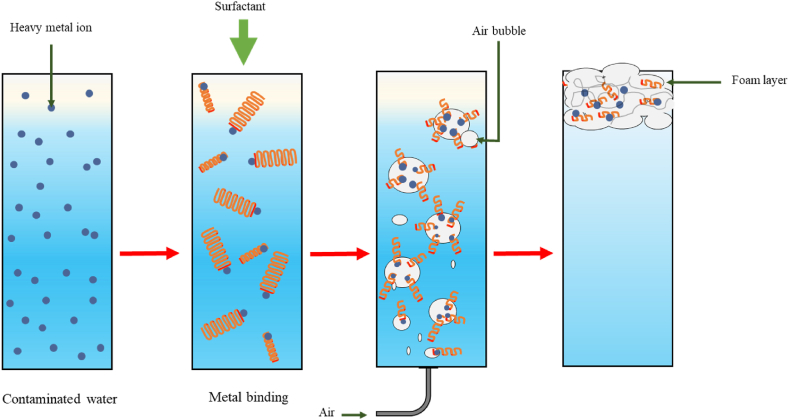
Ion flotation process.

Ion flotation is a wastewater treatment method that utilizes charged molecules called surfactants to attract and remove specific heavy metals. These surfactants, acting as both foaming agents and adsorbents, selectively bind to the target metal ions, forming complexes. Air bubbles are then introduced to the solution, and the metal-surfactant complexes attach to these bubbles, rising to the surface as a foam that can be skimmed off [147] as illustrated in Fig. 8.

Surfactants can be derived from chemical or biological sources, with the latter being preferred due to the environmental benefits [148]. Ion flotation offers several advantages over other methods, including lower energy consumption, reduced sludge production, rapid operation and lower operating costs [149]. However, achieving optimal metal removal efficiency can require significant surfactant [145].

Several studies have demonstrated the effectiveness of ion flotation for heavy metal removal. For example, researchers used microbial surfactants from yeast to treat acidic mine drainage, achieving a 94 % removal rate for iron ions [150]. Another study successfully removed 84 % of cadmium from simulated wastewater using a specific ratio of a typical chemical surfactant dodecyl sulfate (SDS) and a frothing agent [151]. Similar successes have been reported for mercury, copper, and other metals using various collector molecules [152,153].

Applying nano-sized collectors in ion flotation represents a novel technology with the potential for enhanced removal efficiencies and reduced surfactant use. In Ref. [145], this approach achieved removal rates above 95 % removal for lead, cadmium, and copper, showcasing its promise. Ref. [145] also noted that incorporating functionalized graphene oxide (FGO) in the ion flotation process for wastewater treatment provides notable advantages such as high adsorption efficiency, reduced collector usage, recyclability, and the elimination of frothing issues. These findings highlight the effectiveness of ion flotation, particularly with strategically chosen collector molecules, for removing heavy metals from wastewater.

ii. Sorptive or adsorption flotation

Sorptive flotation was derived from the integration of adsorption and flotation. The use of biosorption technology in removing heavy metals from industrial wastewater is a recent development. It offers significant benefits as it economically and efficiently reduces heavy metal ion concentrations to extremely low levels using cost-effective biosorbent materials [154]. This method involves using tiny particle sizes of adsorbents followed by flotation to separate the loaded metal-sorbents [155,156].

Lead ions removal efficiency from wastewater was enhanced by 10 % when sorptive flotation was applied using SDS surfactant and barley husk biosorbent compared to results obtained using only flotation [157]. Copper ions were removed using a cetyl pyridinium bromide (CPB) collector to achieve 97.09 % removal [158]. In his research, a researcher [159] developed an artificial neural network to predict copper ion removal from simulated wastewater using SDS surfactant and sunflower seed husk adsorbent to achieve maximum removal efficiency at 500 cm^3^/min flowrate, 30 g/L,7 g/L, and 30 g/L initial metal, adsorbent, and SDS concentrations [159].

iii. Hybrid flotation

Hybrid flotation is an innovative approach that combines traditional flotation techniques with other separation processes for heavy metal removal. It involves the initial bonding of the metals to a unique bonding agent, which can later be separated from the wastewater stream using specific separation processes. Electrocoagulation and electroflotation [160], electroflotation and filtration [161], membrane microfiltration and flotation [162], ion and adsorbing flotation [163] are recent advances in hybrid flotation [155]. A study [164], explored the use of membranes, zeolite, and specific chemical agents in a hybrid flotation process to purify water and extract valuable metals from industrial effluent. The hybrid system consistently met acceptable standards for residual copper in the water, and the froth collected during flotation contained a substantial amount of recoverable metals, as illustrated in Table 2.

**Table 2:** Use of zeolite particles for removal of some heavy metals from industrial wastewater, adapted from [164].

pH level	Zeolite conc. (g/L)	%Removal efficiency	Cu^2+^ recovered (mg/L)	Fe^3+^ recovered (mg/L)	Mn^2+^ recovered (mg/L)
∼6	4	80.56	1.54	0.28	21.54
∼6.4	8	81.56	0.38	0.14	4.64
6.5	4	81.6	0.85	0.14	9.94
6.5	8	80.76	0.25	0.14	3.25

**Table 3:** Comparison of the technologies, advantages, disadvantages, and innovation.

Wastewater treatment Technologies	Brief description of technology	Advantages	Disadvantages	Factors for high removal efficiency	References
Flotation	- It operates by introducing air bubble flotation into water, causing waste particles to separate and rise to the surface. - It is an alternative to sedimentation in water treatment, the solid-liquid separation process is employed to reduce solid concentrations before granular filtration.	- High efficiency in eliminating contaminants - low operating cost - better water quality - Rapid separation - environmentally friendly - most effective methods for removing biochemical oxygen demand (BOD) and suspended solids (TSS) - highly efficient in removing small particles - Widely adopted as an effective tertiary treatment in the pulp and paper industry. - it demonstrates metal selectivity and offers low retention times.	- High Energy and capital cost - High reagent consumption - Environmental pollution	- retention and residence time of bubbles, particle size, solute content, surface tension, gravity, and the type of reagent used.	[142,143,155,156,[278], [279], [280], [281]]
Coagulation/Flocculation	- It is the destabilization and aggregation of colloids, dissolved particles like heavy metals by utilizing coagulants to form larger aggregates which settles due to force of gravity. - For removing con-taminants and colouring pollutants from contaminated water -	- Simple Process - Low capacity cost - reduced BOD, COD and TOC - low retention time - highly biodegradable - The use of alum will lead to a rise in total solids - It could result in corrosion of equipment and gas pipes due to the SO_4_^2−^ compounds in the sludge - It can remove tiny particles - Removal of color, turbidity and heavy metals and	- Increase in the production of sludge volume. - Generation of concentrated sludge - Expensive chemicals - sludge disposal problem - Not effective in removing tiny oil particles or dissolved oil particles	- dosage and type of coagulant, flocculator retention time, pH, mixing rate, stirring device, Geometry of flocculator. - Environmentally friendly and economically viable flocculants that demonstrate high efficiency in flocculation. - better removal performance when combined with biological treatment or filtration	[[173], [174], [175],[281], [282], [283], [284]]
Adsorption	- It involves atoms, ions, or molecules of a substance adhering to the surface of the adsorbent - The standard approach to assess the adsorption mechanism involves adsorption kinetics and isotherm	- Simple Process - Low oprerating cost - High efficiency - High quality of treated effluent - ability to separate wide range of pollutants - Highly efficient for COD and color removal - It can remove most metals	- Result in fouling - Non-selective methods - Regeneration is costly and leads to material loss - Ineffective with some dyestuffs and some metals. - sludge disposal problem	Adsorbent should possess; - large surface area - porous structures - high adsorption capacity - ability to be regenerated and recycled. - pore walls with functional groups capable of adsorbing heavy metals Right choice of temperature, contact time and adsorbent dosage	[[180], [181], [182],[184], [185], [186], [187], [188], [189], [190]] [193,285,286]
Ion exchange	- Ion exchange is a chemical process an aimed at eliminating undesired dissolved ions from both water - the removal of contaminants from water by replacing them with an ionic substance that is considerably safer	- highly efficient regeneration process - Cheap initial operating cost - Highly efficient in eliminating inorganic ions from water - Easy to operate. - Flexible and suitable for various water treatment processes	- High cost of operation - Expensive regeneration cost - Constructing and operating the equipment can be expensive. - Few metal ions are removed - Resin degradation - They cannot eliminate bacteria	- Development of new ion exchangers is required to reduce the disparity in decontamination factors. - Innovative composites utilizing combinations of both existing and novel ion exchangers - Disposal method for spent ion is required	[8,[204], [205], [206], [207]]
Chemical precipitation	- It involves changing the form of dissolved metal ions into solid particles through the addition of precipitants – .	- Simple and cheap process - Efficient removal method - It can remove most metals - Highly reduced COD - It operates effectively across a wide temperature range	- Large quantity of sludge is generated - Expensive reagent - It involves the use of corrosive chemicals that may pose health hazards	- Thorough evaluation of precipitant selection, dosage and conditions - less harmful chemical should be used to control environmental impact – .	[[213], [214], [215], [216],^218^,^287^]
Photocatalysis	It uses light energy as its sole energy source to produce electron-hole pairs, which are involved in the detoxification of pollutants	- Non-selective and efficient, with the photocatalyst being reusable multiple times - Effective removal of complex pollutants - Cheap operating cost - Environmentally friendly	- Regeneration may be costly - costly source of energy - High energy-intensive and pressure conditions - Not visible for small and medium-sized industries - Low process capacity	Effective selection of the following factors is required; - suitable reactor, pH, degradation concentration, temperature, the charged nature of the pollutant. - Integrating photocatalysis with other wastewater treatment methods	[220,[226], [227], [228], [229]] [287,288]
Electrochemical coagulation (EC)	It is a treatment process that utilizes electrical current to treat and aggregate contaminants, eliminating the need for additional coagulants. - It integrates principles from coagulation, electrochemistry, and flotation.	- Equipment are simple and easy to operate - EC operates without the need for any chemicals, thus eliminating the release of toxic ions. - reduced sludge generation - High removal efficiency of BOD, COD and color - It is capable of treating numerous wastewaters with high toxicity	- High Operation cost at limited electricity - High cost of equipment - High cost of sludge treatment	- Important factors such as electrode polarity, Size of particles, wastewater conductivity, and pH of wastewater should be considered.	[[232], [233], [234], [235], [236],^289^,^290^]
Nanotechnology-based filtration	- utilizes nanoparticles and nanostructured materials to remove heavy metals from wastewater	- High surface area and chemical reactivity leading to increased adsorption rates - rapid, simple and efficient - No need for chemical additives - Minimal production of solid waste - Complete removal of salts, dyes, salts, and mineral derivatives - Effective removal of suspended solids, particles, and microorganisms	- High operation and maintenance costs - Limited flow rates and throughput - Rapid membrane fouling - Reusability of nanoadsorbent is a major problem	- Improving the synthesis and utilization of nanomaterials. - To investigate the consequences of the harmful and long-term effects of nanomaterials on the ecosystem. - Utilizing environmentally friendly and cost-effective nanomaterials.	[[245], [246], [247], [248], [249],^291^,^292^]
Genetic Engineering (GE)	- It is used to enhance the capacity of numerous microorganisms to bioremediate wastewater.	- faster and more effective for wastewater treatment - can manage higher volumes of wastewater flow - High electrical energy - sludge disposal problem - environmentally friendly and Cheaper process	- health hazards on human health - environmental pollution through genetically modified organisms (GMO) – .	- Efforts should be dedicated to addressing pollution problem arising from GMO - Exploring the use of nanoparticles to enhance the efficiency and precision of genetic engineering by facilitating the delivery of genetic material to microorganisms. - modifification of the genes of microorganisms for a more effective bioremediation - Advancements in GE to engineer biosensors capable of detecting environmental pollutants.	[32,[255], [256], [257], [258], [259], [260],[293], [294], [295]]
Microbial Fuel Cells (MFCs)	- directly converting organic waste inside wastewater into electricity - MFCs degrades pollutants via anaerobic oxidation using microbes	- eco-friendly and efficient method. - No harmful by-products generated - Reduced sludge generation - minimal chemical usage and energy requirement - It operates at room temperature with a neutral pH level. - Enhancing sustainability by increasing energy efficiency and lowering operational costs.	- High cost of maintenance - Low rate of production - Slow initial start-up, increased costs, and low efficiency - Capital expenditure (CAPEX) is higher than other methods - Biofouling, Limited power generation, and unstable voltage. - Seasonal variables, like temperature and humidity, impact the performance of MFCs. - MFC process is expensive due to the need for replacement of membrane	- MFCs can be scaled up by enlarging the anodic chamber volume or stacking multiple modular MFCs. - Capex can be reduced by modifying membranes and improving the performance of the cathode and anode	[[266], [267], [268], [269], [270], [271]]

The hybrid flotation system offers a promising, innovative approach that combines the benefits of flotation and several separation techniques while addressing the challenge of sludge production common to the flotation separation process.

#### 4.1.2 Coagulation/flocculation

**Fig. 9:**
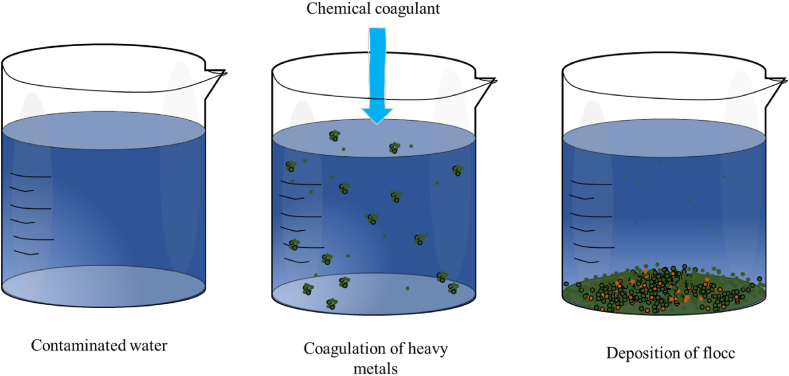
Coagulation/flocculation technology.

Coagulation-flocculation is widely recognized in wastewater treatment due to its cost-effectiveness and efficiency in removing heavy metals [165] (see Table 3). It involves destabilizing and aggregating colloids, dissolved substances, or particles, such as heavy metals, using coagulants to form large aggregates [166]. The aggregates settle under gravity or through alternative means to allow filtration from the bulk liquid in subsequent processes, as illustrated in Fig. 9. Coagulants may be chemically or biologically based. Conventional chemical coagulants employed in coagulation-flocculation processes typically consist of aluminum-based and iron-based compounds, which are needed in substantial quantities for effective treatment and may leave toxic residues in the solution [167]. Plant-derived coagulants are a promising alternative to chemical coagulants since they offer high efficiency, environmental friendliness, and cost-effectiveness in wastewater treatment, and they overcome the problem of toxic leftovers [168,169]. Investigators have identified the Moringa plant, clearing-nut tree, wild cactus, Cassia fistula, and bean seeds as eco-friendly coagulants that have proven effective in removing heavy metals [170,171]. Coagulation technology is mainly employed in treating effluents from the paint industry compared to other methods [172]. Chemical coagulation, on the other hand, does not operate well in low-temperature water, requires expensive equipment with significant maintenance costs, and produces large volumes of sludge with negative impacts on human health. The use of natural coagulants is cost-effective, offers material clarity, and has minimal effect on the pH of the treated water. It is highly biodegradable, which is highly advantageous compared to chemically derived coagulants [[173], [174], [175]]. Recent research developments have focused on optimizing coagulation/flocculation performance, with key parameters including the use of innovative coagulants like organic coagulants [144], biopolymers flocculant and chitosan [176] composite coagulants, hybrid coagulants [177] biocoagulants [178], and modified natural coagulants, which improve heavy metal removal; adjusting mixing speed and time to enhance floc formation and settling; determining optimal temperature ranges for different coagulants and wastewater types; using advanced imaging and particle size analysis to characterize flocs and link their properties to removal efficiency; integrating coagulation/flocculation with other methods such as adsorption, filtration, or membrane technologies for better heavy metal removal; and understanding the kinetics of the coagulation/flocculation process to fine-tune treatment parameters.

In a study conducted by Vishali [172], he evaluated the effectiveness of Cassia fistula as a natural coagulant for treating synthetic water-based paint industry effluent (SPIW). He compared the results with those obtained using a ferrous sulfate coagulant. 5 g of prepared coagulant demonstrated the highest treatment efficiency by achieving 96.15 % color removal and 97.85 % turbidity reduction in the SPIW without altering the initial pH. These results were comparable to those obtained with the chemical coagulant ferrous sulfate. The findings highlighted C. fistula as an effective natural coagulant in treating paint industry wastewater. In a pilot-scale study by Edebali [179], Cassia fistula coagulant was used to treat wastewater from the textile industry. 93.83 % removal efficiency was achieved in a 30 L volume of wastewater, and coagulant dosage of 1.17 mg/L pH levels and coagulant dosage have a significant impact on the coagulation-flocculation process. The Cassia fistula seed gum has a zeta potential of −15.7 mV, indicating its stability and dispersion in aqueous solutions. Utilizing Cassia fistula seed gum for coagulation-flocculation proves to be a viable first treatment for wastewater that could precede membrane or biological treatments while maintaining environmental compliance [179].

#### 4.1.3 Adsorption

**Fig. 10:**
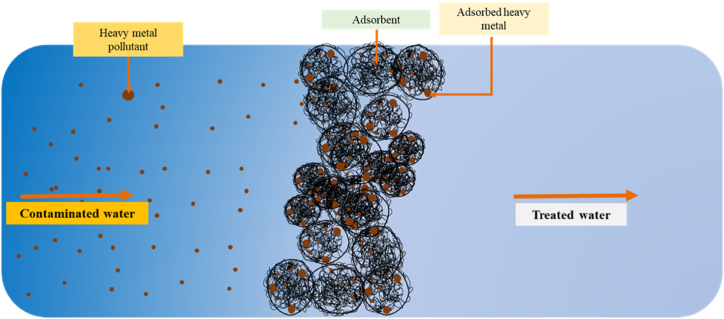
Adsorption technology.

Adsorption is a widely recognized separation technique for wastewater treatment and water purification. The process of adsorption is illustrated in Fig. 10. It has geared attention due to its economic, effective, and versatile design and simplicity of operation compared to other techniques. Researchers have successfully removed heavy metals using this method [[180], [181], [182], [183]]. Adsorbents are usually characterized by high surface area and porous structures for effective adsorption. Biomaterials, natural and waste materials, and membranes have been used as effective adsorbents for water purification processes [[184], [185], [186], [187], [188], [189], [190], [191]]. Heavy metal removal from wastewater is done on the surface of the adsorbents either physically, based on van der Waals force, or chemically, based on covalent bonds formed [192]. The pore walls of selected adsorbents should have significant functional groups that selectively adsorb the heavy metals, have high adsorption capacity, and can be regenerated and recycled [193].

The adsorption capacity (q_e_) of an adsorbent in g^−1^ is determined by [194].

23 $$q_{e}=\left(\frac{C_{o}-C_{f}}{W}\right)\times V$$

Where; C_o_ and C_f_ are the initial and final concentrations of adsorbate in ppm,

W is the amount of adsorbent in g and.

V is the volume of solution in L.

Adsorption isotherms (AI) show the relationship between the adsorbed heavy metal ions and the adsorbent at equilibrium [61]. AI is the graphical representation that depicts the relationship between the amount of adsorbate on the adsorbent and its concentration in the solution at equilibrium. The Langmuir model describes adsorption at active adsorbent sites in a monolayer adsorption process, while the Freundlich model shows multilayer or heterogeneous surface adsorption. The models predict the adsorbents' adsorption capacity, whether mono- or multi-layered.

Adsorption kinetics (AK) shows the mechanisms involved in metal adsorption using pseudo-first-order and pseudo-second-order models to predict the optimal conditions needed in batch adsorption processes. The AK explains the speed at which adsorption happens and are extensively employed to analyze dynamic adsorption behaviors and uncover mass-transfer mechanisms in batch systems. Choosing the right models to analyze sorption data is challenging because of the numerous available kinetic models. However, understanding that adsorption rate constants must be independent of operating parameters and that the equilibrium equation should be a special kinetic equation case can help select the appropriate kinetics for the sorption process [195]. AK can be examined through different models and mechanisms with common ones including.

i) Pseudo-First-Order Model (PFO): This model assumes that the occupation rate of adsorption sites is proportional to the number of unoccupied sites. The linear form of the equation is:

24 $$log\left(q_{e}-q_{t}\right)=\log\;q_{e}-\frac{k_{1}}{2.303}t$$

Where $q_{e}$ is the amount of adsorbate at equilibrium, $q_{t}$ is the amount at time t, and k_1_ is the rate constant.

ii) Pseudo-Second-Order (PSO) Model: The adsorption rate is proportional to the square of the number of unoccupied sites. The linear form of the equation is:

25 $$\frac{t}{q_{t}}=\frac{1}{k_{2}q_{e}^{2}}+\frac{t}{q_{e}}$$

Where $k_{2}$ is the rate constant for the second-order model.

iii) Intraparticle Diffusion Model: Accounts for the diffusion of adsorbate within the pores of the adsorbent. It can be described by:

26 $$q_{t}=k_{1}t^{0.5}+C$$

Where k_1_ is the intraparticle diffusion rate constant, and C is a constant.

However, recent developments in kinetic models have refined these models' assumptions, mathematical formulations, and applications, leading to a better understanding and prediction of adsorption processes. Researchers have developed non-linear versions of the PFO and PSO models to avoid errors caused by linear transformations, resulting in a more precise fit for experimental data [196,197]. Recent advancements include incorporating time-dependent rate constants into the PFO model to better handle systems with variable adsorption rates and adding temperature dependence to the PSO model using Arrhenius-type expressions to account for temperature variations [198]. Additionally, several advanced kinetic models have been developed to overcome the limitations of traditional models. These include the Avrami Kinetic Model [199], which addresses complex adsorption mechanisms and surface heterogeneity, and Fractional Order Kinetics [200], which extends traditional integer-order kinetics to fractional orders for improved flexibility and fitting in specific adsorption systems.

Adsorption and coagulation-flocculation techniques are two widely employed technologies for removing dyes from industrial wastewater. The ease of operation, cost-effectiveness, and high efficiency in addressing dye pollutants make them preferred over other methods. Both techniques significantly treat wastewater, particularly in industries where dye removal is critical to environmental compliance [201,202]. Among other adsorbents, activated carbon is renowned for its capacity to adsorb organic materials using low-cost adsorbents. A study by Alabi [203] explored the adsorption kinetics and mechanism of methylene blue and Congo red by activated Cassia fistula adsorbent. The study highlighted the influence of physicochemical factors, including initial solution pH, dye concentration, adsorbent dosage, and temperature, on the adsorption capacity.

#### 4.1.4 Ion exchange

**Fig. 11:**
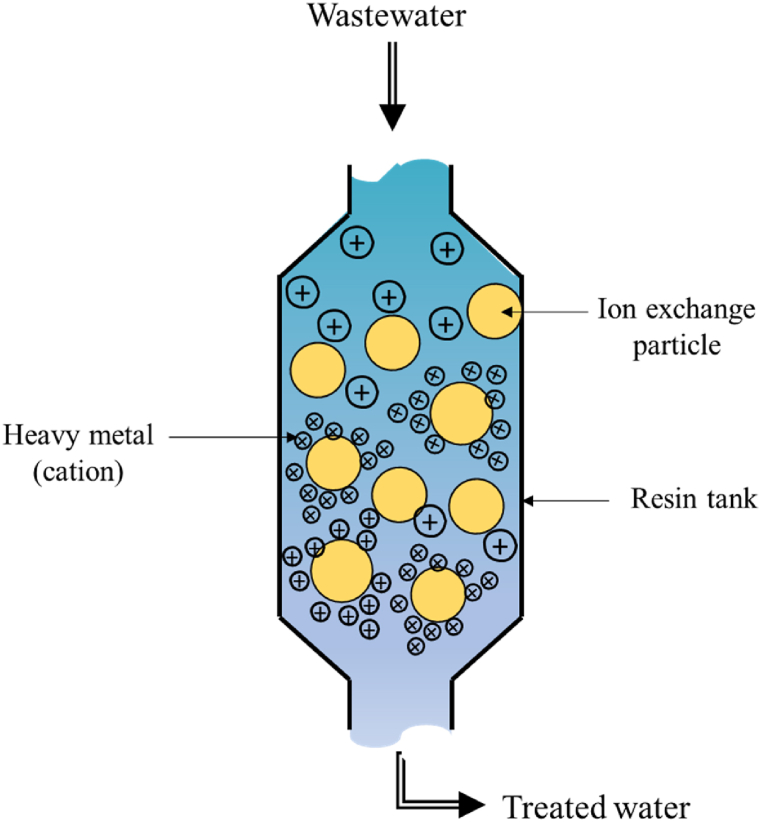
Ion exchange technology.

Ion exchange is a reliable technology for removing heavy metals from industrial wastewater. It involves using a solid material known as the ion exchanger or resin bead, which can swap unwanted heavy metal ions in the wastewater with more favourable ions in its structure. The resin beads contain essential functional groups that act as magnets by selectively attracting and holding unto specific heavy metal ions as they flow through the water. Fig. 11 gives a pictorial representation of the process. Several studies have demonstrated the effectiveness of ion exchange in heavy metal remediation [[204], [205], [206], [207]]. Research conducted in 2019 used ion-exchange resin sachets and micro-XRF core-scanning to rapidly assess heavy metal pollution in a suspected polluted area in central Taiwan [205]. The study found that the ion-exchange resin sachets effectively concentrated the heavy metal cationic species. This allowed for precise assessment of elemental concentrations using the fast and non-destructive XRF-CS technique [205]. Another study reviewed various methods for eliminating heavy metal ions from wastewater, including ion exchange [206]. His process works by replacing hazardous metal ions with non-toxic, environmentally friendly ones, suggesting that ion exchange could be a viable solution for heavy metal contamination in wastewater. Additionally, another research investigated organic-inorganic ion exchange materials for removing heavy metals from water, highlighting their high efficiency and the technological simplicity of the ion exchange process [207]. Furthermore, research published in 2019 evaluated the technical feasibility of ion exchange methods for removing various heavy metals from aqueous media, presenting the chemical pretreatment of low-cost biosorbents [208].

Ion exchange offers several advantages as a heavy metal removal technology. It is highly selective and can be tailored to target and remove specific heavy metals from the wastewater stream, making it a highly precise solution. Furthermore, operating and maintaining an ion exchange system is relatively simple and cost-effective [209]. Another significant advantage is that the ion exchange resins can be reused. These resins can be regenerated and used for wastewater treatment, minimizing operational expenses and environmental impact. However, ion exchange has its limitations. It is not effective for removing heavy metals at deficient concentrations; in these cases, the ion exchange resin may not have enough capacity to capture all the metal ions [209].

Furthermore, ion exchange resins degrade over time, reducing their effectiveness and requiring periodic replacement. The resin's regeneration process produces waste brine containing concentrated heavy metals, necessitating treatment before disposal. Further research is necessary to enhance the efficiency of ion exchange and expand its applicability in wastewater treatment. Optimal pH levels vary depending on the specific metals and resins used [144], . Adequate contact time between the resin and the metal-containing solution is essential to achieve maximum removal efficiency. The metal removal efficiency is also influenced by the amount of resin used relative to the solution volume. Generally, slower flow rates enhance removal efficiency, and higher temperatures can increase the ion exchange rate, though they may also affect resin stability. Not all ion exchangers are suitable for all metal ions [210], and pretreatment may be necessary if the wastewater contains oil or grease [211]. In his study, ref. [212] recommend maintaining an alkaline pH using a suitable base, like NaOH, using strong acid cation exchange resin, and ensuring sufficient residence time for effective ion exchange.

#### 4.1.5 Chemical precipitation

**Fig. 12:**
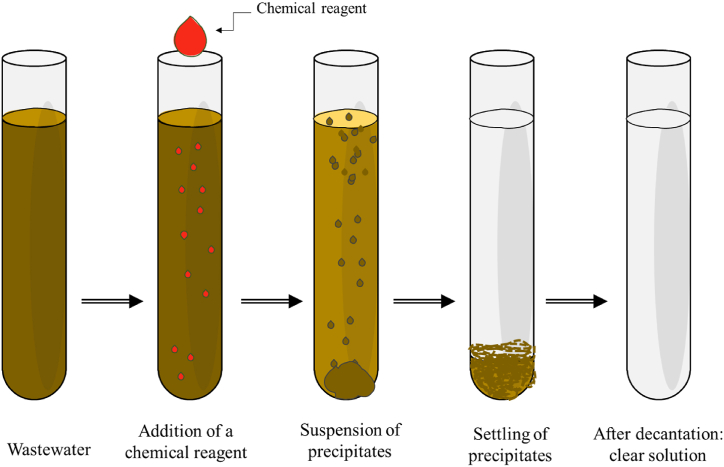
Chemical precipitation.

Chemical precipitation is a widely used technology for removing heavy metals from industrial wastewater. It involves changing the form of dissolved metal ions into solid particles by adding precipitants, facilitating their sedimentation and subsequent removal from the water [213] as illustrated in Fig. 12. Several studies have explored chemical precipitation's effectiveness, advantages, limitations, and applications in heavy metal removal from wastewater [[214], [215], [216]].

A study [217] demonstrated the use of synthetical magnesium hydroxy carbonate for the chemical precipitation of heavy metals from wastewater, achieving removal efficiencies above 99.9. Another study reviewed the removal of heavy metal ions from wastewater, highlighting the potential of chemical precipitation as a reversible reaction to replace undesirable metal ions with environmentally friendly ions, making it a promising technology for heavy metal removal [206]. Research conducted in 2020 focused on the chemical precipitation of heavy metals from wastewater, identifying the precipitate composition mainly composed of Fe_2_O_3_, V_2_O_5_, and Cr_2_O_3_, which can be recycled as secondary raw material for the metallurgical industry [218]. A study in 2021 explored the removal of heavy metal ions using Fe_3_O_4_ particles, co-precipitation, high-gravity technology, and grafting, showcasing the versatility and effectiveness of chemical precipitation methods in heavy metal removal [219].

Chemical precipitation has several advantages as a heavy metal removal technology. It is highly selective, allowing for removing specific heavy metals from wastewater. It is also relatively simple to operate and maintain, making it a cost-effective solution for heavy metal removal. Additionally, ion exchange resins can be regenerated and reused, reducing costs and environmental impact.

However, chemical precipitation also has some limitations. It may not be effective for removing heavy metals in very low concentrations, as the ion exchanger may not have sufficient capacity to bind to the metals. Additionally, ion exchange resins may degrade over time, reducing their effectiveness and requiring replacement. Generating large volumes of sludge during chemical precipitation requires proper disposal, which can present additional challenges and costs.

Despite these limitations, chemical precipitation has been proven effective in removing heavy metals from wastewater, offering a selective and cost-efficient solution. By changing dissolved metal ions into solid particles, this method facilitates their removal from water, contributing to environmental protection and human health.

However, for high-level performance of chemical precipitation, the process hinges on several key factors: increasing the pH value of the treatment system [16], choosing the right precipitant tailored to specific metals [144], and employing advanced dosing systems to achieve the correct balance. Additionally, advanced filtration or chemical treatment methods and reducing or oxidizing agents may be necessary. Regular maintenance and calibration of equipment and monitoring systems are crucial for high performance. Ref. [15] noted that one of the most effective methods of chemical precipitation is using hydroxides or sulfides, which offer a higher degree of metal reduction over a wide pH range in a shorter time.

### 4.2 Modern technologies for heavy metals removal from industrial wastewater

#### 4.2.1 Photocatalysis

Photocatalysis represents an innovative catalytic oxidation method that harnesses light energy as its sole energy source to produce electron-hole pairs, which are involved in detoxifying pollutants [220]. This novel, environmentally friendly technology finds extensive application in purifying water from organic and inorganic contaminants such as heavy metals due to its remarkable versatility in environmental remediation material [26]. It originated from studies on hydrogen for environmental applications and research on imitating photosynthesis [221].

Generally, photocatalysts are substances used in photocatalysis that often have semiconductor structures. They are made up of two parts: the valence band (VB) and the conduction band (CB). When the semiconductor photocatalyst material is exposed to light (usually ultraviolet or visible light), electrons (e^−^) in the valence band, which is the bottom part of the semiconductor, may jump to the conduction band, which is the upper part of the semiconductor given sufficient energy, thereby leaving a positively charged hole (h^+^) on the VB. The energy required for the transition can be attained if the energy of the photon in the incident light or light source is greater than or equal to the bandgap energy between the semiconductor valence band (VB) and the conduction band (CB)as shown in the equation [26]. [222].

27 $$\text{Photocatalyst}\;\text{VB}\;\left(e^{-}\right)+h\nu \;\left(\text{UV}\;\text{or}\;\text{visible}\;\text{light}\right)\;\to \;h^{+}\left(\text{VB}\right)+e^{-}\left(\text{CB}\right)$$

**Fig. 13:**
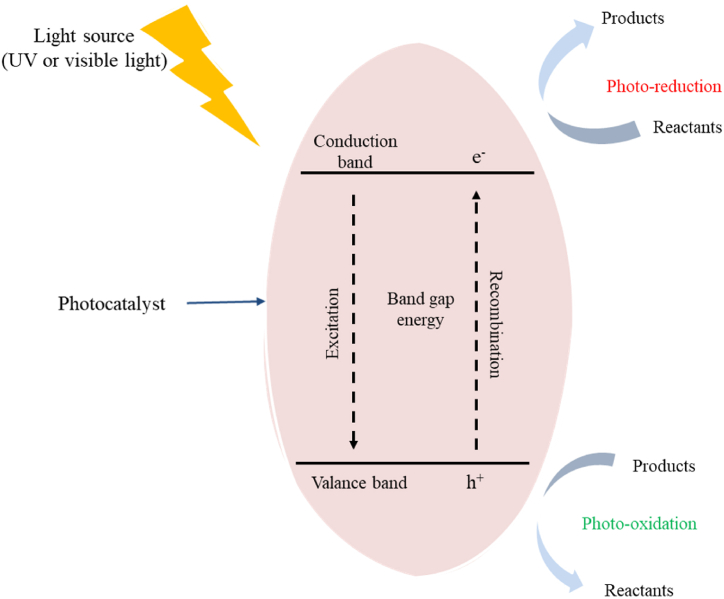
Photocatalysis.

The generated electron-hole pairs (h^+^ and e^−^) can migrate to the surface of the photocatalyst to participate in a series of reduction-oxidation reactions for converting heavy metals to less harmful forms, as illustrated in Fig. 13.

The study of chromium metal removal from industrial effluents is gaining more attention due to its widespread applications across numerous industrial and technological sectors and toxicity. A review on heavy metal treatment using photocatalysis highlighted successful studies of hexagonal chromium photoreduction using immobilized TiO_2_ under a UV light in 32 h [26]. A rapid decrease in the concentration of Cr (VI) ions was observed at a pH level of 2, the solution treated with TiO_2_ coated with hydroxyl aluminum tricarboxymonoamide phthalocyanine (AITCPc) was subjected to visible light. This reduction was facilitated by the presence of 4-chlorophenol (4-CP) as a sacrificial donor to prevent the photobleaching of the AITCPc. The titania (TiO_2_) dye was excited, and an electron was introduced into the conduction band to facilitate the reduction of Cr(VI) ions [223]. This photoreduction resulted in converting most Cr (VI) ions into Cr (III) ions, and a small fraction being reduced to Cr atoms as illustrated in equation 28, 29, 30, 31) [224,225].

28 $$\text{Cr}\left(\text{VI}\right)+e^{-}\;\to \;\text{Cr}\left(V\right)$$

29 $$\text{Cr}\left(V\right)+e^{-}\;\to \;\text{Cr}\left(\text{IV}\right)$$

30 $$\text{Cr}\left(\text{IV}\right)+e^{-}\;\to \;\text{Cr}\left(\text{III}\right)$$

31 $${\text{Cr}}_{2}O_{7}^{2-}+6e^{-}+14H^{+}\;\to \;2{\text{Cr}}^{3+}+7\;H_{2}O\;\left(E^{0}=+1.33\;V\right)$$

Despite its innovative potential, the practical application of photocatalysis is still limited by the low photocatalytic efficiency, high cost and poor stability [226]. To enhance their photocatalytic performance, researchers have explored methods such as doping, creating nanocomposites, integrating photocatalysis with other wastewater treatment technologies, and creating mathematical models to optimize reaction conditions parameters [[227], [228], [229]]. Furthermore, photocatalysts with low cost, high stability, low environmental impact, and ease of recovery and reuse should be considered [229].

Optimizing various parameters can significantly enhance the removal of heavy metals and other contaminants for highly efficient photocatalysis performance. This includes developing new materials with higher catalytic activity and stability or modifying existing materials to improve their photocatalytic properties [26]. Increasing the surface area of photocatalysts boosts the number of active sites available for reactions, while adjusting pH levels can impact the process's effectiveness [230]. Although higher temperatures generally increase reaction rates, excessively high temperatures may deactivate the photocatalyst. Key characteristics such as band gap energy, crystallinity, and defect density also play critical roles in performance. Extending light absorption into the visible spectrum through sensitization with dyes or other substances, and ensuring longer exposure times, can lead to higher degradation rates. However, ref. [219] suggested that, photocatalysis can have low throughput, be pH-dependent, and become inefficient in the presence of different metals. Also, to improve the efficiency, ref. Suggested that recent modification strategies such as vacancy engineering, heterojunction construction, and facet engineering should be explored [231].

#### 4.2.2 Electrochemical coagulation

**Fig. 14:**
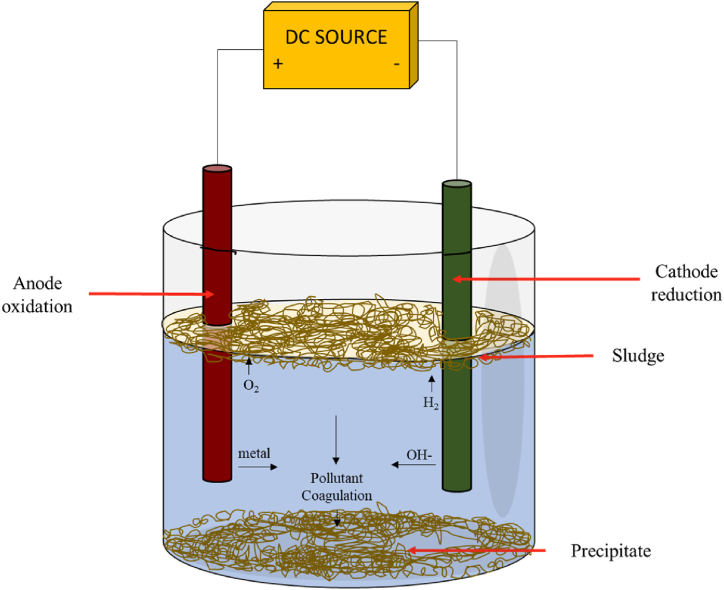
Electrochemical coagulation.

Conventional methods for removing heavy metals from water have limitations such as incomplete metal removal, the need for constant monitoring, and the use of expensive equipment. Electrochemical coagulation (EC) is a promising solution to address these limitations [[232], [233], [234], [235]]. Electrochemical coagulation is an electrochemical technique that eliminates several water contaminants, including metals, organic impurities, dyes, pigments, colloidal solids, and soluble inorganic pollutants. The process employs sacrificial electrodes like metals (Fe, Al) and Al alloys [236] to generate a charge at the interface between the electrode and the fluid. This charge leads to the coagulation and precipitation of suspended particles within the fluid [237]. The process of electrochemical coagulation is illustrated in Fig. 14.

A study by Vasudevan [238] described electrochemically assisted coagulation technology for chromium removal from water, using zinc as the anode and galvanized iron as the cathode. The study investigated the impact of various parameters, including pH, current density, chromium concentration, temperature, adsorption kinetics, and isotherms. The study showed an optimal chromium removal efficiency of 96.5 % at a current density of 0.2 A/dm^2^ and a pH of 7.0.

Electrochemically assisted coagulation operates by generating metallic ions electrochemically. These ions undergo hydrolysis near the anode, producing active intermediates that can effectively destabilize finely dispersed particles in the water. This method offers advantages such as high particulate removal efficiency, a compact treatment facility, and the potential for complete automation. Electrocoagulation minimizes the need for additional chemicals influencing sludge production [238]. While this EC effectively eliminates numerous pollutants, it may encounter practical constraints when targeting newly emerged biodegradable and soluble organic pollutants.

For electrochemical coagulation (EC) to perform at a high level in the removal of heavy metals from wastewater, several key parameters must be optimized. The choice of electrode material, such as Fe, stainless steel or Al, significantly impacts coagulation efficiency, while the shape of the electrodes affects pollutant removal efficiency. Although EC is generally effective at ambient temperatures, certain conditions may necessitate temperature adjustments. Optimizing the flow rate is crucial for maximizing removal efficiency while maintaining manageable treatment times. Regular maintenance and periodic cleaning of electrodes are essential to sustain high performance. Maintaining an optimal pH range, typically between 6 and 8, is vital for effective removal. The optimal current density depends on the specific wastewater composition and desired removal efficiency. Refs. [239,240] suggested that more research is needed to validate the operational parameters for scaling up the process, as the values differ depending on the effluents. Ref. Indicated that most studies in the literature have been conducted at the laboratory scale using synthetic solutions, a pilot plant scale experiment is required to explore its potential for treating industrial wastewater.

Ref. [241] explored the combination of electrochemical coagulation (EC) and adsorption (EC-AD) for heavy metal removal and found that hybrid technologies are more effective and economical compared to individual adsorption or electrocoagulation methods. Ref. [242] supported this conclusion, noting that the combined strategies improve the removal of chemical oxygen demand (COD), inorganic salts, antibiotics, dyes, colloidal particles, and turbidity pollutants. Ref. [243] achieved high removal efficiencies using EC with Fe and Al electrodes and ozonation, reporting 100 % removal for V and Pb, 99.35 % for Cr, 99.51 % for Fe, 87.31 % for Ni, 99.83 % for Cu, 99.65 % for Zn, 88.46 % for TOC, and 76.28 % for COD. Ref. [244] demonstrated a 99.88 % removal efficiency for Pb(II) using a solar-integrated EC-AD method. However, ref. [241] concluded that while EC-AD processes have proven effective in controlled settings, further research is needed in actual wastewater treatment plants or field sites to assess these technologies' scalability, reliability, and cost-effectiveness in practical scenarios.

#### 4.2.3 Nanotechnology-based filtration (NF)

**Fig. 15:**
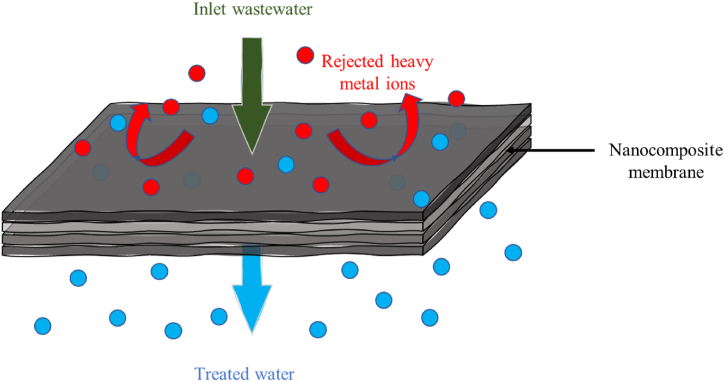
Nanofiltration.

Nanotechnology-based filtration is a promising heavy metal technology that utilizes nanoparticles and nanostructured materials to remove heavy metals from wastewater [[245], [246], [247], [248], [249]]. This technology effectively removes a wide range of heavy metals, including lead, copper, and nickel. There are different types of nanocomposites categorized by their matrix materials; this includes metal nanocomposites (Ni/Al_2_O_3,_ Al/CNT, Al/CNT, Fe-Cr/Al2O_3_, Mg/CNT, Co/Cr, Fe-MgO), Ceramic nanocomposites (Al_2_O_3_/TiO_2_, Al2O_3_/SiO_2_, Al_2_O_3_/CNT, Al_2_O_3_/SiC, SiO_2_/Ni) and Polymer nanocomposites (polymer/layered silicates, Thermoplastic/thermoset, polyester/TiO_2_, polymer/layered polymer/CNT, double hydroxides) [250,251]. A research paper employed nanoparticle-infused membranes for extracting heavy metals from wastewater. The researchers found that the nanoparticle-based membranes could effectively remove heavy metals, with removal efficiencies of up to 99 % [217]. Another study used nanofibrous membranes to remove heavy metals from wastewater. The researchers found that the nanofibrous membranes could effectively remove heavy metals, with removal efficiencies of up to 98 % [252]. NF presents numerous advantages compared to conventional methods for heavy metal removal; nanoparticles and nanostructured materials possess a notably high surface area-to-volume ratio, enabling efficient adsorption of heavy metals. Moreover, these materials can be functionalized with diverse functional groups, augmenting their capacity for heavy metal adsorption. Nonetheless, NF encounters certain constraints. For instance, producing nanoparticles and nanostructured materials can be costly and necessitate specialized equipment for handling. Furthermore, their recovery and recycling processes may pose challenges, potentially elevating technology costs. A nanofiltration process is illustrated in Fig. 15.

The efficiency and performance of nanotechnology-based filtration (NF) systems depend on several critical parameters which include: the number and size of pores on the membrane surface is a crucial factor for membrane performance [253]. The size and shape of nanoparticles also play a significant role, affecting the reactivity, surface area, and filtration efficiency. Additionally, membrane thickness and porosity influence the flow rate and pressure drop across the membrane. Cost-effectiveness, disposal costs, energy consumption, and maintenance of the nanomaterials are important economic considerations. Research by Ref. [43] highlights the need to reduce energy costs for easier NF membranes (NFM) commercialization, improve technology for timely fouling detection, and address the impact of repeated membrane cleaning on its lifespan. Ref. [247] noted that sustainable fabrication of NFM can be achieved through interfacial polymerization and concluded that further research will explore their effectiveness in heavy metal removal. Furthermore, there is a demand for more environmentally friendly and cost-effective nanoparticles with exceptional performance in NFM. Ref. [254] warned that excessive use of certain antibacterial nanoparticles, like graphene derivatives, can affect the ecological balance of water and soil, emphasizing the need for future research to understand the hazards and behavior of nanoparticles.

#### 4.2.4 Genetic engineering

Genetic engineering is an innovative approach that harnesses the potential of genetically modified microorganisms (GMMs) and genetically engineered organisms (GEOs) to effectively remove heavy metal ions from polluted water [32,[255], [256], [257], [258], [259], [260]]. This technique aims to manipulate genes to boost plants' heavy metal tolerance and accumulation [261]. The most common strategy for increasing heavy metal tolerance is to enhance antioxidant activity, which can be achieved by overexpression of genes involved in antioxidant machinery. Imagine tiny shields protecting plant cells. Genes involved in the uptake, translocation, and sequestration of heavy metals can be introduced and overexpressed in target plants1 to increase heavy metal accumulation. These genes encode metal ion transporters, including ZIP, MTP, MATE, and HMA family members [262], responsible for the uptake and transport of heavy metals in plants. Genetic engineering can also promote the production of metal chelators in plants. Metal chelators act as metal-binding ligands to improve heavy metal bioavailability, promote heavy metal uptake and root-to-shoot translocation, and mediate intracellular sequestration of heavy metal ions in organelles1. By overexpression of genes encoding natural chelators, heavy metal uptake and translocation can be improved.

A study investigated the potential of genetic transformation to make plants more resistant to heavy metals [259]. This involved tweaking the plants' metabolic pathways related to stress defence, leading to more efficient phytoremediation (removal of pollutants by plants). Another study focused on genetically engineered microbes designed to tackle toxic heavy metals, exploring both the potential and challenges of this method [32].

This bioremediation method offers several advantages over traditional techniques. It is highly cost-effective and environmentally friendly for heavy metal removal [260,263]. GMMs and GEOs can be tailored to target specific heavy metals, reducing the need for broad-spectrum treatments and minimizing environmental impact. Additionally, these engineered organisms can be highly efficient in accumulating heavy metals, leading to faster and more thorough cleanup.

However, there are limitations to this approach. The complex mechanisms involved in detoxifying and storing heavy metals require manipulating numerous genes, which can be time-consuming and often unsuccessful. Additionally, genetically modified plants face strict regulations and difficulty obtaining approval for field testing, hindering their real-world application.

For high-level performance in genetic engineering, several key factors are crucial for success and efficiency, which include selecting an appropriate vector (virus, plasmid, etc.), which is vital for delivering genetic material into target cells [264]. Ensuring the gene is mutation-free and properly sequenced and using a suitable promoter for efficient transcription in the host organism are essential. Consistent gene expression stability over time is also critical. Ref. [264] explained that research is needed to optimize bioremediation using plants and microorganisms, identifying the most effective organisms and conditions. Ref. [265] noted that introducing genes into fast-reproducing microorganisms can enhance efficiency, though using genetically modified microorganisms (GMMs) in practical fields is debated due to potential health risks. However, Ref. [32] suggested that minimizing these risks involves discovering processes to manage direct risks and assessing their impact for comprehensive risk evaluation.

#### 4.2.5 Microbial fuel cells (MFCs)

**Fig. 16:**
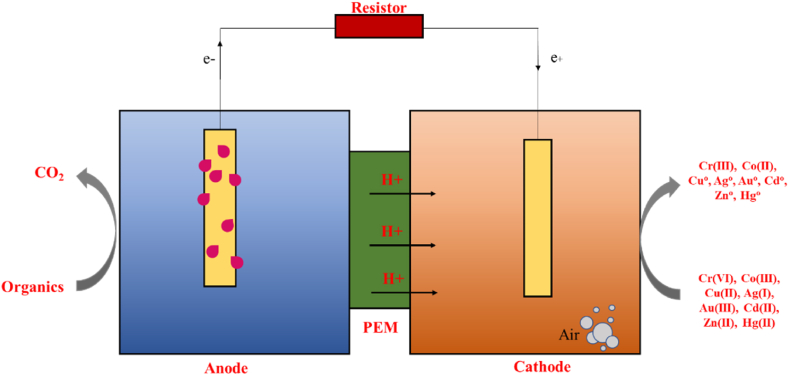
Microbial fuel cell technology.

Microbial fuel cells (MFCs) are innovative biological treatment technologies that can generate electricity while treating wastewater contaminated with heavy metals [[266], [267], [268], [269], [270], [271]]. The operation of MFCs (Fig. 16) for heavy metal removal involves harnessing the metabolic activity of electroactive bacteria to facilitate the reduction and removal of heavy metal ions from wastewater. The MFC consists of an anodic compartment where bacteria break down organic matter, releasing electrons and protons. These electrons are then transferred to the cathode through an external circuit, while protons migrate through a proton exchange membrane (PEM). Heavy metal ions act as electron acceptors in the cathode compartment, undergoing electrochemical reduction and eventual removal from the system.

Recent research is exploring the full potential of MFCs for heavy metal removal. Studies have shown that MFCs can simultaneously remove these contaminants and generate electricity [272]. Furthermore, they can target specific heavy metals based on their properties, allowing for efficient removal and recovery [273]. Research is ongoing to optimize MFC performance. Studies have investigated how different types of organic matter and plant life within the MFC system can enhance the removal of specific heavy metals like zinc and nickel [274].

One of the advantages of microbial fuel cell technology over other technologies is that MFCs not only remove heavy metals but also generate electricity, making them an energy-efficient solution. They operate at normal temperatures and pressures, minimizing energy needs and operational costs. MFCs offer a green technology with the potential to recover valuable resources from wastewater.

Successfully translating MFC technology from lab settings to large-scale applications remains a challenge. Variations in the microbial community within the MFC can affect its performance. Carefully designing and engineering MFCs is necessary for seamless integration with existing wastewater treatment infrastructure. The effectiveness of MFCs in removing heavy metals depends on factors like the specific type and concentration of the metal, as well as the design of the MFC system itself. These highlight the limitations of this technology.

Several factors are essential for optimal performance of Microbial Fuel Cells (MFCs). These include adapting microbes to the specific MFC conditions and substrates used, increasing electrode surface areas to enhance microbial attachment and electrochemical reactions, and ensuring proper spacing between the anode and cathode to reduce internal resistance. Maintaining a neutral [275] to slightly acidic pH by optimizing electron transfer mediators and operating conditions further improves MFC efficiency [276]. Continuous systems often offer more stable performance, and high-redox potential metal ions can be reduced at the cathode [37]. However, challenges remain, such as the inability to treat all metal ions and the need for further research on effective substrates for large-scale wastewater treatment applications [35,277].

## 5 Critical analysis and future trends

Mismanagement of industry-related waste and industrial activity growth endangers the environment, human health, and economic sectors that depend on the availability and quality of natural resources from water bodies. Wastewater contamination causes significant environmental harm, including ecosystem degradation and dangers to human life, among other negative impacts. Industrial effluent discharge profoundly impacts ecosystems, deteriorating water quality and disrupting normal rhythms. Heavy metals, dyes, and other organic pollutants contribute significantly to this pollution. In the succeeding section, the flaws of wastewater treatments have been highlighted to include incomplete removal of contaminants (i.e., mainly from conventional wastewater treatment technology, inadequate removal of nutrients (i.e., nitrogen and phosphorus from wastewater), energy-intensive processes (i.e., leading to high energy consumption), chemical dependency (i.e., significant reliance on certain chemicals), byproducts of treatment processes (i.e. leading to limited resilience to climate change), inadequate treatment of industrial and hazardous waste (i.e., emanating from specified purification methods leading to the emergence of other pollutants such as organic matter, nutrients, and pathogens), and regular issues in developing countries (i.e., limited public awareness and engagement). Some of these flaws have serious implications after treatment; for example, inadequate removal of nutrients can lead to eutrophication, harmful algal blooms, and disruptions to aquatic ecosystems. Chemical dependency leads to the generation of disinfection by-products and chemical residues in treated effluent. Byproducts of treatment processes could lead to further environmental risk [247].

Chemical precipitation is a popular method for treating waters with high heavy metal concentrations due to its simplicity, effectiveness, low capital cost, and suitability for automation. However, it may not consistently achieve residual metal concentrations below regulatory limits, necessitating further advanced treatment. Coagulation-flocculation processes face similar challenges. Electrochemical cells (EC) provide selective ion removal and recovery without extra chemicals but are energy-intensive. Membrane separation offers high efficiency but is hampered by high costs and membrane fouling. For waters with low metal ion content, ion exchange and adsorption are used. Ion exchange involves significant investment and issues with resin costs, selectivity, and reusability, while adsorption is cost-effective and easy to manage but struggles with adsorbent regeneration. Photocatalysis, although effective, is energy-intensive and can pose health and environmental risks. Microbial Fuel Cells (MFCs) are environmentally friendly and efficient but face high maintenance costs and low production rates. Combining several treatment processes, like adsorption, precipitation, and membrane filtration, can improve removal efficiency.

Developing novel and environmentally acceptable ways to remove heavy metal ions from wastewater is a promising future approach. As a result, additional research into the treatment of various heavy metal complexes is proposed, which includes the following: Energy consumption and environmental sustainability of treatment processes [249], potential for resource recovery from wastewater, technological innovation drives and modification, evaluation of existing environmental regulations and policies, evaluation of existing regulations and standards compliance which will serve as the framework for future technological improvement in wastewater treatment, evaluation of environmental footprint of wastewater treatment byproducts and processes, capital and operational costs of wastewater treatment technologies, political will-power to enforce policies on potential industrial investors, and resilience of wastewater treatment infrastructure to climate change impacts.

Future research should focus on creating cost-effective, efficient, and environmentally friendly materials and methods for wastewater treatment. Evaluating each treatment approach from laboratory to pilot scale is essential to assess its practicality. Additionally, leveraging political will to engage industrial leaders in resource recovery and reuse, adopting onsite and community-scale treatment solutions, and incorporating advanced technologies like blockchain, IoT sensors, and AI can enhance wastewater management. Improvements in real-time monitoring, optimization, and predictive maintenance, along with considering climate change resilience, are crucial. Developing portable and adaptable treatment plants can also help manage increasing wastewater volumes. Overall, the goal is to develop environmentally sound and commercially viable solutions to combat heavy metal pollution and protect water bodies.

## CRediT authorship contribution statement

**T.E. Oladimeji:** Writing – review & editing, Writing – original draft, Supervision, Resources, Formal analysis, Conceptualization. **M. Oyedemi:** Writing – original draft, Resources, Formal analysis. **M.E. Emetere:** Writing – review & editing, Formal analysis. **O. Agboola:** Writing – review & editing, Formal analysis. **J.B. Adeoye:** Writing – review & editing, Formal analysis. **O.A. Odunlami:** Formal analysis.

## Data availability statement

No data was used for the research described in the article.

## Declaration of competing interest

The author declares that there is no conflict of interest regarding the publication of this manuscript. In addition, the authors have completely observed the ethical issues, including plagiarism, informed consent, misconduct, data fabrication and/or falsification, double publication and/or submission, and redundancy.
